# A Systematic Review of the Epidemiologic Literature Assessing Health Outcomes in Populations Living near Oil and Natural Gas Operations: Study Quality and Future Recommendations

**DOI:** 10.3390/ijerph16122123

**Published:** 2019-06-15

**Authors:** Alison M. Bamber, Stephanie H. Hasanali, Anil S. Nair, Sharon M. Watkins, Daniel I. Vigil, Michael Van Dyke, Tami S. McMullin, Kristy Richardson

**Affiliations:** 1 Disease Control and Environmental Epidemiology Division, Colorado Department of Public Health and Environment, Denver, CO 80246, USA; daniel.vigil@state.co.us (D.I.V.); mike@mv2sci.com (M.V.D.); tmcmullin@cteh.com (T.S.M.); kristy.richardson@state.co.us (K.R.); 2 Bureau of Epidemiology, Pennsylvania Department of Health, Harrisburg, PA 17120, USA; c-shasanal@pa.gov (S.H.H.); annair@pa.gov (A.S.N.); shawatkins@pa.gov (S.M.W.)

**Keywords:** oil and natural gas, hydraulic fracturing, fracking, unconventional oil and gas, environmental health, epidemiology, systematic literature review

## Abstract

A systematic method was used to review the existing epidemiologic literature and determine the state of the scientific evidence for potential adverse health outcomes in populations living near oil and natural gas (ONG) operations in the United States. The review utilized adapted systematic review frameworks from the medical and environmental health fields, such as Grading of Recommendations, Assessment, Development and Evaluations (GRADE), the Navigation Guide, and guidance from the National Toxicology Program’s Office of Health Assessment and Translation (OHAT). The review included 20 epidemiologic studies, with 32 different health outcomes. Studies of populations living near ONG operations provide limited evidence (modest scientific findings that support the outcome, but with significant limitations) of harmful health effects including asthma exacerbations and various self-reported symptoms. Study quality has improved over time and the highest rated studies within this assessment have primarily focused on birth outcomes. Additional high-quality studies are needed to confirm or dispute these correlations.

## 1. Introduction

The United States has significantly increased its capacity for oil and natural gas (ONG) development through the technological advancements of directional drilling and hydraulic fracturing, with natural gas production reaching a high in 2017 and 2018 [1]. In 2016, more than two-thirds of the 977,000 producing ONG wells in the U.S. used these technologies to access energy reserves in shale and tight oil sands [2]. In places like the Colorado Front Range and Dallas-Fort Worth, Texas, ONG operations are occurring directly alongside population growth. It is estimated that 17.6 million people in the U.S. live within 1 mile of an active ONG well [3].

There currently exists limited research and conflicting scientific information on the health risks for those living next to these operations. The industry surrounding ONG expanded faster than evidence-based epidemiologic research could respond [4,5]. Early community health assessments and surveys of health symptoms in people living near ONG operations raised concerns about the potential chemical hazards, including exposures to air and water pollution [6,7,8]. Additional studies pointed to non-chemical stressors, including psychosocial stress, from living near ONG operations [9,10,11]. These early hypothesis-generating studies gave way to a growing body of observational epidemiologic literature that has quantified associations between residential proximity to ONG operations and the potential for certain adverse human health effects. Several review articles published within the last five years summarize this literature [5,12,13,14].

Our study is the first of its kind to systematically review the entirety of existing epidemiologic literature on the associations between living near ONG development and the potential for harmful health effects. We weigh the level of evidence for each health outcome and aim to present a clear assessment of the methodological rigor, study strengths, and weaknesses, to identify approaches to future research. The scholarship published to date varies in the types of ONG operations studied, the populations of interest (e.g., based on their geography, time period, or demographic characteristics), the health outcomes measured, and the quality of the methods used. While Saunders and colleagues do raise important methodological concerns about many of the articles they review [14], no existing review addresses study quality in a systematic way. In research on the health effects of potential environmental contaminants, where randomized controlled trials are neither ethical nor appropriate, study quality, or certainty in the study aligning with its stated objectives, is integral to interpreting scientific results and extrapolating them for regulatory and other science-based decisions.

The need for public health scientists to systematically evaluate the body of a literature base for an important issue, with limited resources, is necessary to assist in science-based regulatory decision making. Often, these issues are not entirely characterized and may include multiple chemical stressors (which are typically unknown) and variable health outcomes. The current established systematic review frameworks focus on an in-depth evaluation of the toxicological and epidemiological literature for a specific chemical and/or health outcome, however, this approach is unable to be applied directly to the epidemiological literature surrounding ONG development. Therefore, we have adapted these approaches to better answer this environmental health question.

**Figure 1:**
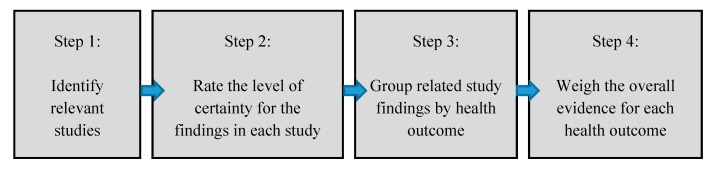
Steps in the current systematic review of epidemiologic literature.

The steps used to conduct the review were adapted from various established systematic review frameworks for the medical and public health fields, including as Grading of Recommendations, Assessment, Development and Evaluations (GRADE) [15] and Meta-analyses Of Observational Studies in Epidemiology (MOOSE for observational studies) [16], and emerging methods in environmental health as outlined by the Navigation Guide [17], and Office of Health Assessment and Translation (OHAT) [18] guidance (Figure 1). Each study was evaluated using 14 study evaluation questions to assess the level of certainty in, or scientific plausibility of, the study findings. The overall weight of evidence was determined for each health outcome separately. This review is not intended to replicate any previous frameworks nor is it to be the single word on study quality in this area of research. Our aim is to be objective and transparent, in a way that can be understood by community members, government and non-government public health and environmental officials and policymakers.

## 2. Materials and Methods

### 2.1. Scope of Analysis

**Figure 2:**
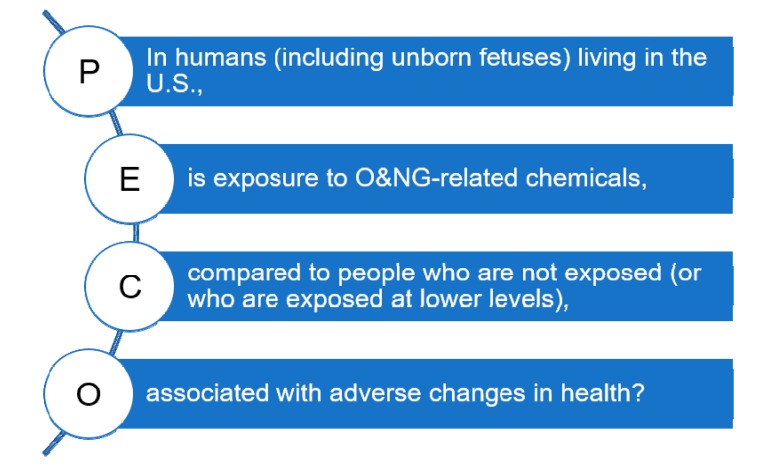
Populations, exposures, comparators, and outcomes (PECO) statement.

The scope of this literature review is defined by a PECO (populations, exposures, comparators, and outcomes) question [19]: “In humans (including unborn fetuses) living in the U.S., is exposure to chemicals emitted from ONG operations, compared to people who are not exposed (or who are exposed at lower levels), associated with adverse changes in health?” (Figure 2). Unborn fetuses were included as a population of interest to account for the possibility of ONG activities affecting fetal development within the mother’s womb. The term “oil and natural gas operations” (or development) was defined to include all upstream processes involved in the extraction of ONG resources using any combination of vertical drilling, directional/horizontal drilling, and hydraulic fracturing to access energy reserves from conventional and unconventional geologic formations. This review does not include studies evaluating mid- and downstream processes. Since October 2011, the majority of new ONG wells in the U.S. overall have been hydraulically fractured horizontal wells, typically referred to as unconventional wells [2]. Study authors will often use a variety of these terms, and the distinction between conventional and unconventional wells—in source rock, depth, or drilling technique—is muddled in practice [20]. We sought to look across a range of comparators since exposures to ONG-associated chemicals occur along a continuum and it may not always be clear what the pathway of exposure is, how far that pathway reaches, or whether multiple exposure pathways produce synergistic effects on health [5,19]. We then considered whether any and all adverse changes in health occur with these exposures. While it is plausible that ONG may impact health through indirect pathways such as income (e.g., from monetary gains from leasing land or mineral rights), or investment in community infrastructure such as healthcare services [10,21,22], indirect effects were not included in this paper.

The PECO question informed our exclusion criteria and studies were excluded if one or more of the following five criteria were met: (1) exposure to ONG chemicals was not directly measured in, or estimated for, study subjects (i.e., excluded studies focused on indirect health effects including community stressors such as degradation of rural life, sexually transmitted infections from newly arrived young male workers, and traffic accidents from increased heavy truck traffic); (2) the study failed to quantify associations between exposures and a specific health outcome (i.e., excluded studies did not measure odds ratios, relative risk, etc.); (3) the study did not include original data or observations (e.g., review articles, commentaries); (4) the study did not define ONG operations to include any or all processes associated with the upstream development and production of ONG, including but not limited to horizontal drilling and hydraulic fracturing; or (5) the study did not take place in the U.S.

### 2.2. Data Search

**Figure 3:**
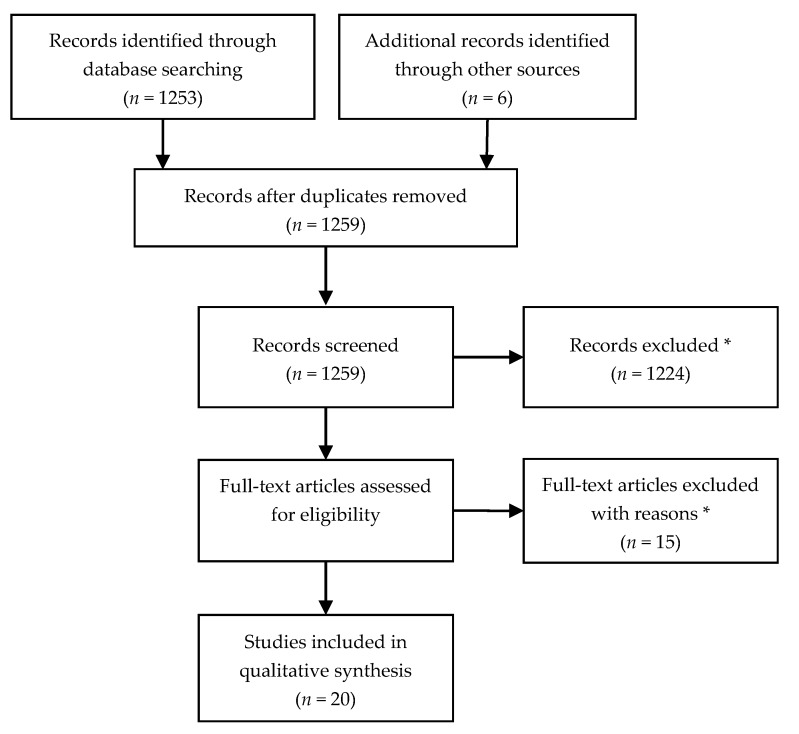
Preferred Reporting Items for Systematic Reviews and Meta-Analyses (PRISMA) flow diagram for study inclusion. * Exclusion criteria is detailed within the methods.

PubMed was the primary research database used to obtain articles. We identified relevant records using the following PubMed search terms: ((“Oil and Gas Industry”[Mesh] OR “Natural Gas”[Mesh]) AND (epidemiolog* or symptom*)) OR ((oil OR natural gas) AND (epidemiolog* OR health OR symptom*) AND (unconventional OR drilling OR shale OR coal OR production OR development) NOT (“Occupational Health”[Mesh] OR “Animal Experimentation”[Mesh]) AND (“2013/01/01”[PDAT]: “2018/10/01”[PDAT])) AND Humans[Mesh]. We verified that no relevant study was published before 2013, and any studies published after our search date of October 1, 2018 were not included in the assessment. In total, 1253 articles were returned by the search and all were screened for eligibility (Figure 3). Review articles, risk assessments, and included studies were also screened for references and identified six additional studies. The majority of articles (98%) did not meet our study inclusion criteria because they were related to the fields of environmental engineering, geology, hydrology or biomedical topics such as plant-based oil extracts/lipids. We kept the search terms broad in an effort to capture the wide variety of terminology that has been used within the interdisciplinary ONG health effects field.

### 2.3. Level of Certainty Rating and Level of Evidence Conclusions for Individual Studies

**Figure 4:**
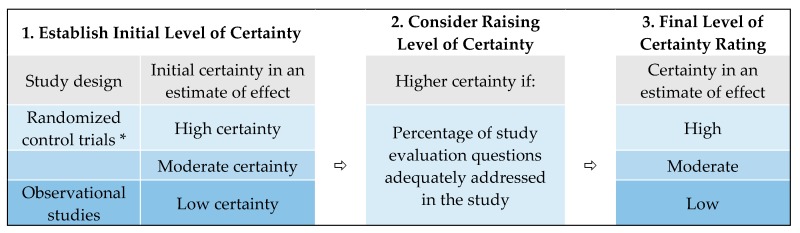
The approach used for developing level of certainty ratings for each study outcome. * No randomized control trials were identified in this review.

A modified systematic review framework was used to rate the level of certainty (or the certainty in an estimate of effect) for each health outcome (Figure 4). We developed our framework based on established methods of systematic reviews for the medical, public health and environmental health fields. These frameworks incorporate, either explicitly or implicitly, most of Bradford Hill’s criteria for causation such as studies with specificity and biological plausibility and that were temporal and consistent [23]. We consulted these classic criteria to develop a meaningful scope of review (as reflected in the PECO question) and determine criteria for study certainty and weight of evidence [24].

We rated study findings as having low, moderate, or high certainty that the estimated effect was close to that of the true effect. The findings of observational epidemiologic studies were initially ranked as low certainty and were upgraded according to fourteen (14) study evaluation questions that assessed various domains (Table 1). These criteria were based on established frameworks which specify the domains, questions, or study limitations used to evaluate individual studies for use in a systematic review [17,18,25,26,27]. We categorized the study evaluation questions into five groups: population and sample, exposure, health outcomes, confounders, and reporting. Two or more authors reviewed each study evaluation question with a yes-or-no response for each study (Supplementary Tables S1–S20). Conflicting responses were resolved through discussion and additional review of the study. Studies with greater than 50% “yes” answers (i.e., 8 “yes” answers out of 14) were considered for potential upgrade of their findings to moderate certainty; studies with greater than 75% “yes” answers (i.e., 11 “yes” answers out of 14) were considered for potential upgrade to high certainty [28]. All findings of each study were ascribed the same level of certainty after evaluations were complete.

We derived weight-of-evidence conclusions using standards outlined in GRADE [29], the Cochrane Handbook [30], and developed by the Institute of Medicine [31]. For each health outcome, relevant findings from individual studies were grouped and evaluated to derive one of the following weight-of-evidence levels: substantial, moderate, limited, mixed, failing to show an association, or insufficient (Table 2).

## 3. Results 

Twenty (20) studies met our criteria of a human health epidemiologic study evaluating the potential health effects associated with living near ONG operations in the United States (Table 3, Supplementary Table S21). Weight-of-evidence conclusions were developed for a total of 32 different health effects, and ranged from insufficient evidence to limited evidence (Table 4).

Across all health outcomes, four of the 20 studies received a moderate level of certainty rating. All others received a rating of low certainty. The majority of the studies were retrospective cohort (six studies) or ecological (six studies) study designs. There were five cross sectional studies, two nested case controls, and two case-controls. The average score across all studies was 6, with a score range from 2 to 9 (Supplementary Table S22).

### 3.1. Birth Defects and Birth Outcomes

This review identified nine studies comprising 12 low to moderate certainty findings that identified the relationship between women who lived near ONG operations and the likelihood that their child was born with birth defects or other types of adverse health outcomes at birth. 

Two studies evaluated birth defects (congenital heart defects, oral clefts, and neural tube defects) in infants of mothers who lived at varying proximities to ONG development during pregnancy [32,33]. These low-certainty studies resulted in insufficient evidence to determine if living near ONG operations during pregnancy is associated with birth defects since there was only one study per outcome.

Eight studies evaluated adverse birth outcomes [32,34,35,36,37,38,39,40]. These studies examined commonly used indicators of infant health status such as preterm birth, gestational age, Apgar score, birth weight, infant mortality, and fetal death. Overall, there are conflicting findings across studies resulting in either mixed or insufficient evidence of adverse birth outcomes associated with living near ONG operations during pregnancy (Table 4). Three of the eight studies and their findings were upgraded to a moderate level of certainty rating due to strength in their study designs that reduced risk-of-bias [35,37,38]. These studies demonstrated both positive and null associations for multiple health outcomes. All three were retrospective cohort studies that demonstrated evidence of a dose-response relationship and included a valid exposure surrogate as taken from a point location. All other studies were ranked as low certainty because of limitations within the study design or missing key elements. For example, most studies failed to adequately quantify exposure either directly, or through a proxy/surrogate estimate. In many cases, this measure of exposure was limited to either presence or absence of wells in a county or was solely proximity-based. Although some studies calculated inverse distance-weighted well counts, they failed to quantify other metrics such as well development phase or total natural gas volume [39]. 

Birth outcomes have received the most scholarly attention for this topic, due to the relatively easy access to birth certificate or birth health records data, and the ability to pinpoint exposures to ONG operations during the 40-week gestation period [36]. While the overall evidence is rated as mixed or insufficient for various outcomes, the most recently published studies on ONG and birth outcomes have used innovative methodologies that improve or alleviate some of the weaker assumptions in early work. For example, Hill in 2018 took advantage of the little assumed difference between pregnant women living near permitted but not yet drilled wells and those living near active wells to define a better comparison or control group [37]. Additionally, three of the four moderate certainty studies evaluated birth outcomes and have identified positive associations between living near ONG operations and these adverse health outcomes.

ONG operations can emit volatile organic compounds (VOCs) into the air and contribute to increased particulate matter 10 micrometers or less in diameter (≤PM_10_) during upstream development activities. Some of these VOCs have the potential to cause developmental effects in test animals following high levels of exposure—generally at much higher levels than what has been observed for individual VOCs at ONG operations [41]. Systematic reviews of a broad set of data have identified positive associations between maternal exposures to fine particulate matter in ambient outdoor air pollution in urban areas and adverse birth outcomes. Other studies have documented adverse developmental and reproductive health outcomes in animals exposed to ONG-related chemicals used as fracturing fluids in the hydraulic fracturing process [42,43,44,45]. Although these substances may be released from operations, the exposure concentrations and complete routes of exposure have not been well characterized.

### 3.2. Cancer

We identified seven low certainty study outcomes from three studies that assessed the relationship between living near ONG operations and the likelihood of developing cancer [46,47,48]. The studies examined various types of both adult-onset and childhood cancers. Specifically, they looked at the incidence of cancers of the urinary bladder and thyroid, leukemia, all childhood cancers, childhood leukemia (and specifically acute lymphocytic leukemia), childhood non-Hodgkin’s lymphoma, and childhood central nervous system tumors. Overall, the weight of evidence is insufficient for all but one of the cancer outcomes since there is only one study for each. There is mixed evidence for childhood leukemia owing to conflicting study findings.

None of the three cancer studies and their findings were upgraded to a moderate level of certainty rating. Two of the studies were ecological, conducted at the county level in Pennsylvania, and did not control for potential confounding variables [46,47]. For example, it is probable that there are social characteristics of county populations (e.g., race or ethnicity, occupation, smoking status, etc.), differing access to medical care and screening, and other environmental exposures (e.g., major roadways, particularly in a place like Allegheny County where Pittsburgh is located) that would explain some of the study findings. Fryzek et al. also incorrectly interpreted their standardized incidence ratio results, as has been noted by Saunders et al. [14]. McKenzie et al. used a case-control design to study childhood cancers in rural Colorado [48]. However, their data source was exclusively the state’s cancer registry and therefore there was no comparison group made up of children without cancer. Additional research on this topic might consider incorporating a more appropriate comparison group from household surveys [49]. For studies of cancer, it is crucial for researchers to consider what would be an appropriate time frame from exposure to ONG operations to the potential development of cancer. ONG operations began in earnest in the late 2000s in Pennsylvania, but Fryzek et al. used data only through 2009; this truncated period between community exposure and cancer endpoint is a major limitation [47]. As noted elsewhere [50], the study period was not matched to the theoretical lag period or latency period for adult carcinogenesis.

ONG operations may release chemicals into the air and water, such as benzene, polycyclic aromatic hydrocarbons, and diesel exhaust [51]. Although long-term exposure to these substances, such as benzene, may increase the risk of developing certain types of cancer, the development of cancer is complex because many other non-environmental influences, such as genetics and lifestyle behaviors, also contribute to cancer risk.

### 3.3. Respiratory Health Outcomes

There were three low to moderate rated health outcomes from six studies evaluating the associations between living near ONG and respiratory health effects [52,53,54,55,56,57]. A single moderate certainty study with one study outcome indicated a limited weight of evidence for an association with asthma exacerbations [56]. The current literature provides a link between regulated air pollutants (ozone and particulate matter) and lung, heart disease and other respiratory health effects [58]. The influence, specifically, of ONG contributing to respiratory health outcomes is not fully understood, particularly within the context of other behavioral/lifestyle influences (e.g., smoking) exacerbating the deleterious effects of air pollutants. Additionally, there may be many other environmental sources of emissions for air pollutants including vehicles and wildfires.

Five other low-rated studies evaluated the occurrence of respiratory effects (various self-reported symptoms and hospitalizations) and found conflicting evidence for both categories. The two hospitalization studies used ecological study design, which is limited since the estimation of exposure is based on an average in the population. The three other studies documented self-reported symptoms. Health outcomes were not determined by a medical provider.

### 3.4. Neurological Health Outcomes

We identified four studies that assessed the relationship between living near ONG development and the likelihood of neurological health effects [52,53,55,57]. Three studies identified self-reported neurological symptoms (Elliott et al. [52]: severe headaches, dizziness; Rabinowitz et al. [55]: neurologic problems, severe headache/migraine, dizziness/balance problems, depression, difficulty concentrating/remembering, difficulty sleeping/insomnia, anxiety/nervousness, seizures; Tustin et al. [57]: migraine headache, fatigue) and yielded a limited weight of evidence for a null association with neurological health effects. The other outcome, neurological hospitalizations, had insufficient evidence, with only one positive study published [53]. VOCs are known to produce neurological effects, such as central nervous system damage, headaches, dizziness, visual disorders, loss of coordination, and memory impairment in test animals and humans [59].

### 3.5. Other Health Outcomes

We found limited evidence of a positive association between general multiple self-reported symptoms and living near ONG development, with two studies assessing this relationship [52,57]. The two studies however characterized symptoms differently: Elliott and her colleagues combined feeling stress, fatigue, muscle or joint pain, or any other health symptom into a “general health symptom” grouping [52]; while Tustin and his co-authors found significant effects only when at least two of the three symptoms they considered—chronic rhinosinusitis, migraine, and fatigue—were experienced jointly [57].

Two epidemiologic studies evaluated a variety of indicators of psychological well-being, including depression, anxiety and sleep disturbances [60,61]. Measures of mental health are not necessarily a result of direct exposure to substances emitted from oil and gas operations but could be indirectly associated with non-chemical environmental stressors such as noise, light, odors, or social stress of living near a hotly debated, politicized, and potentially risky industry. For example, studies have shown associations between living in areas with increased noise and traffic, such as by airports, with increased psychological symptoms [62,63,64,65].

There was mixed evidence for self-reported dermal symptoms, self-reported psychological symptoms, and cardiovascular hospitalizations. Other health effects, including neurological and all hospitalizations, diagnosed sleep disturbances, and self-reported cardiovascular symptoms, had insufficient evidence due to a single low-rated study per outcome. There was a demonstrated lack of evidence (no association) for gastrointestinal self-reported symptoms. Three studies evaluated self-reported dermal symptoms, such as rash, irritation, burning, itching, and hair loss, in relation to ONG in Pennsylvania, resulting in mixed evidence [52,55,61]. Skin-related health effects may be possible due to direct exposure to soil or water. However, the routes of exposure to ONG-related chemicals were not well characterized in these studies and encounters with other skin irritants were not documented, making it difficult to interpret these conclusions.

## 4. Discussion

In this paper, we summarized the observational epidemiologic literature on the health effects of populations living near ONG operations and assessed the methodological rigor of the studies published to date. Specifically, we used a modified systematic review framework, adapted from GRADE, the Navigation Guide, and guidance from OHAT, to determine the level of certainty that the study findings represent the true effect of exposures to ONG-related substances, and to make overarching weight-of-evidence determinations for a variety of health outcomes.

The strength of our review lies in its transparency and objectivity. We adapted previous systematic review guidelines to make the criteria for evaluating studies as clear as possible. We considered a wide variety of study evaluation questions to represent those domains. Our review framework can also be applied to other research questions in environmental health. For researchers, policymakers, and public health practitioners, this type of review can swiftly help elucidate key findings and gaps in the knowledge base that need to be addressed.

We found 20 published epidemiologic studies that evaluate potential associations between ONG operations and health outcomes. These studies assessed 32 different health outcomes ranging from self-reported symptoms to confirmed disease diagnoses. Since only a few outcomes were covered by multiple studies, there was insufficient weight of evidence for most health outcomes. We found studies of populations living near ONG operations provide limited evidence (modest scientific findings that support the outcome, but with significant limitations) of harmful health effects including asthma exacerbations and various self-reported symptoms. For all other health outcomes, we found conflicting evidence (mixed), insufficient evidence, or in some cases, a lack of evidence of the possibility for harmful health effects.

There are important limitations to our approach. First, it is not a meta-analysis as the current line of inquiry, including different exposure measures (and surrogates), health outcomes, and geographic/geologic locations, is not suited to conducting a meta-analysis. Second, although we clearly stated our criteria for upgrading a study to a moderate or high level of certainty ranking, the number of study evaluation questions and the ranking cutoffs may still be viewed as arbitrary since Rooney et al. (2016) compares these systematic review methods and notes that the scoring of studies may be influenced by the number of elements and may not account for the differences in relative importance across the risk of bias domains [66]. Study certainty is difficult to quantify, but we used a quantifiable framework and did not allow factors such as media coverage or other publicity (positive or negative) to color our ranking system.

The majority of findings from the studies were ranked as low certainty, primarily due to limitations of the study designs that make it difficult to establish clear links between exposures to substances potentially emitted directly from ONG operations and the health outcomes evaluated. These limitations are inherent to observational epidemiologic studies and include indirect exposure measurements, confounding bias, and subjective methods to determine health outcomes. The field of environmental health incorporates these types of studies along with exposure and risk assessments to inform public health and policies. In addition to these factors, differences in the observational epidemiologic study types (e.g., retrospective cohort, case-control, ecological) make it difficult to compare results across studies with various health outcomes. These epidemiologic studies may also reflect the interactions of non-chemical or chemical stressors that may or may not be related to ONG operations that can contribute to adverse health outcomes in a population. Study quality has improved in recent years with better exposure measures and more thorough methods to account for possible confounders.

Although these observational epidemiologic studies alone are not sufficient to determine causality, they provide helpful information to direct further investigation into the public health implications of ONG activity near residential areas. Taken together, these studies make it clear that the identities and exposure levels of substances people are exposed to when living, working, or going to school near ONG development have not been well characterized. Epidemiologic studies that include more controlled designs with direct measurement of exposure and diagnosed health outcomes are needed to confirm or dispute the associations published in the literature. Incorporating a health impact assessment framework within an epidemiologic study may be useful. One such framework, developed by the Agency for Toxic Substances and Disease Registry (ATSDR) can be used to assess the health impacts of multiple chemicals and stressors [67].

Additionally, we have little empirically driven understanding of the factors (biological, geological, meteorological, and social) that drive ONG-related exposure patterns and vulnerability to such exposures. For example, there may be regional differences across the U.S., with varying technological controls or regulatory environments. Researchers should integrate community members [68,69,70] and concepts of health equity and environmental justice [69] into their research approaches. They should also consider using policy as a starting point rather than the conclusion in order to evaluate policies and ONG industry practices that have been implemented thus far (e.g., setback distances, number of wells drilled per well pad, etc.). Having an understanding and familiarity with the populations at risk for health effects from ONG development across states and regions within states is also important to prioritize evidence-based health-protective policy interventions and to improve public health prevention strategies [52,68,69,70,71].

ONG regulatory policy has not been informed by robust epidemiologic research literature. Now, 15–20 years since the widespread application of hydraulic fracturing and horizontal drilling in states as diverse as Colorado, Pennsylvania, Texas, and Kansas, the epidemiologic literature on the potential health effects of ONG operations is still inadequate to definitively guide policy, as evidenced by the mainly low certainty and conflicting studies reviewed here. Regulators and policymakers, then, should work with public health researchers to pose specific questions that need to be answered, and partner with public health officials to evaluate the public’s concerns. Public health officials should continue to monitor health concerns in areas with substantial ONG operations through centralized data collection and analysis. Multi-state collaborations should be considered to collect consistent data from differing oil and gas basins across the United States with the aim to more comprehensively evaluate the potential for adverse health effects.

## Figures and Tables

**Table 1:** Key study evaluation questions to determine the level of certainty ratings for health outcomes.

Study Evaluation Questions
Population and Sample
1. Does the control group match the exposed group?
2. Is the sample generalizable to the population of interest?
3. Did the study a priori quantify sample and power?
4. Were missing data addressed and tested?
Exposure
5. Was exposure directly measured and quantified?
6. Was the exposure or proxy/surrogate of exposure measured from a point location?
7. Does the proxy/surrogate adequately estimate exposure?
8. Was there a temporal relationship between exposure and outcome?
Health Outcomes
9. Was the health outcome determined by a medical provider?
10. Was a dose-response relationship seen in any outcome?
Confounders
11. Did the study design or analysis account for important confounding and modifying variables?
12. Did the study design or analysis adjust or control for other environmental exposures that were anticipated to bias results?
13. Were sensitivity analyses attempted for population, outcome, or exposure?
Reporting
14. Did the study conclusions match the results?
Final level of certainty rating: Low/Moderate/High

**Table 2:** Weight-of-evidence determinations.

Evidence Level	Definition
Substantial	Strong scientific findings that support an association between oil and gas exposure and the outcome, with no credible opposing scientific evidence.
Moderate	Strong scientific findings that support an association between oil and gas exposure and the outcome, but these findings have some limitations.
Limited	Modest scientific findings that support an association between oil and gas exposure and the outcome, but these findings have significant limitations.
Mixed	Both supporting and opposing scientific findings for an association between oil and gas exposure and the outcome, with neither direction dominating.
Failing to show an association	Body of research failing to show an association—indicates that the topic has been researched without evidence of an association; is further classified as a limited, moderate or substantial body of research failing to show an association.
Insufficient	The outcome has not been sufficiently studied.

**Table 3:** Summary details of epidemiologic studies included in this systematic review.

First Author	Year	Title	Publication	State	Study Type	Health Finding Category	Positive Associations	Null Associations	Level of Certainty
**Busby [34]**	2017	There’s a World Going on Underground—Infant Mortality and Fracking in Pennsylvania	Journal of Environmental Protection	Pennsylvania	Ecological	Birth outcomes	Early infant mortality	NA	Low (3)
**Casey [35]**	2016	Unconventional Natural Gas Development and Birth Outcomes in Pennsylvania, USA	Epidemiology	Pennsylvania	Retrospective cohort	Birth outcomes	Preterm birth and high-risk pregnancy ^a^	Apgar score, small for gestational age, term birth weight	Moderate (9)
**Casey [60]**	2018	Associations of Unconventional Natural Gas Development with Depression Symptoms and Disordered Sleep in Pennsylvania	Scientific Reports	Pennsylvania	Case-control and cross-sectional	Self-reported symptoms and diagnoses	Depression symptoms (self-reported)	Disordered sleep (diagnoses)	Low (6)
**Currie [36]**	2017	Hydraulic Fracturing and Infant Health: New Evidence from Pennsylvania	Science Advances	Pennsylvania	Retrospective cohort	Birth outcomes	Low birth weight, decreased birth weight, decreased score on infant health index	NA	Low (5)
**Elliott [52]**	2018	A Community-based Evaluation of Proximity to Unconventional Oil and Gas Wells, Drinking Water Contaminants, and Health Symptoms in Ohio	Cross-sectional	Ohio	Cross-sectional	Self-reported symptoms	General symptoms (stress, fatigue, muscle or joint pain, any other self-reported health symptoms)	Respiratory, neurological ^b^, dermal, gastrointestinal symptoms (self-reported)	Low (6)
**Finkel [46]**	2016	Shale Gas Development and Cancer Incidence in Southwest Pennsylvania	Public Health	Pennsylvania	Ecological	Cancer	Urinary bladder cancer	Thyroid cancer, leukemia	Low (2)
**Fryzek [47]**	2013	Childhood Cancer Incidence in Pennsylvania Counties in Relation to Living in Counties with Hydraulic Fracturing Sites	Journal of Environmental Medicine	Pennsylvania	Ecological	Cancer (child)	Central nervous system tumors	All childhood cancer incidence and leukemia	Low (2)
**Hill [37]**	2018	Unconventional Natural Gas Development and Infant Health: Evidence from Pennsylvania	Journal of Health Economics	Pennsylvania	Retrospective cohort	Birth outcomes	Low birth weight, decreased term birth weight, premature birth small for gestational age, Apgar score less than 8	Gestation periods	Moderate (9)
**Jemielita [53]**	2015	Unconventional Gas and Oil Drilling is Associated with Increased Hospital Utilization Rates	PLOS ONE	Pennsylvania	Ecological	Hospitalizations	Cardiology and neurology hospitalizations	Hospitalizations for various medical categories, including pulmonary hospitalizations	Low (7)
**Ma [33]**	2016	Time Series Evaluation of Birth Defects in Areas with and without Unconventional Natural Gas Development	Journal of Epidemiology and Public Health Reviews	Pennsylvania	Interrupted time series	Birth defects	NA	Birth defects prevalence	Low (5)
**McKenzie [32]**	2014	Birth Outcomes and Maternal Residential Proximity to Natural Gas Development in Rural Colorado	Environmental Health Perspectives	Colorado	Retrospective cohort	Birth outcomes and birth defects	Congenital heart defects and neural tube defects	Oral clefts, preterm birth ^+^, term low birth weight ^+^, decreased term birth weight ^+^	Low (6)
**McKenzie [48]**	2017	Childhood Hematologic Cancer and Residential Proximity to Oil and Gas Development	PLOS ONE	Colorado	Case-control	Cancer (child)	Childhood acute lymphocytic leukemia	Childhood non-Hodgkin’s lymphoma	Low (8)
**Peng [54]**	2018	The Health Implications of Unconventional Natural Gas Development in Pennsylvania	Health Economics	Pennsylvania	Ecological	Hospitalizations	Pneumonia hospitalizations	Hospitalizations for acute myocardial infarction, chronic obstructive pulmonary disease (COPD), asthma, upper respiratory infections	Low (6)
**Rabinowitz [55]**	2015	Proximity to Natural Gas Wells and Reported Health Status: Results of a Household Survey in Washington County, Pennsylvania	Environmental Health Perspectives	Pennsylvania	Cross-sectional	Self-reported symptoms	Dermal and upper respiratory symptoms (self-reported)	Lower respiratory, cardiovascular, gastrointestinal, neurological symptoms (self-reported)	Low (7)
**Rasmussen [56]**	2016	Association Between Unconventional Natural Gas Development in the Marcellus Shale and Asthma Exacerbations	JAMA Intern Med.	Pennsylvania	Nested case-control	Respiratory diagnoses	Asthma exacerbations	NA	Moderate (8)
**Stacy [38]**	2015	Perinatal Outcomes and Unconventional Natural Gas Operations in Southwest Pennsylvania	PLOS ONE	Pennsylvania	Retrospective cohort	Birth outcomes	Decreased birth weight and small for gestational age	Premature birth+	Moderate (8)
**Steinzor [61]**	2013	Investigating Links Between Shale Gas Development and Health Impacts Through a Community Survey Project in Pennsylvania	New Solutions	Pennsylvania	Cross-sectional	Self-reported symptoms	Throat irritation, sinus problems, nasal irritation, eye burning, persistent cough, frequent nose bleeds, loss of sense of smell, severe headaches, skin rashes, swollen painful joints symptoms (self-reported)	Joint pain, sleep disturbances, shortness of breath, forgetfulness, sleep disorders, feeling weak and tired, increased fatigue, lumbar pain, muscle aches or pain, diarrhea symptoms (self-reported)	Low (3)
**Tustin [57]**	2016	Associations between Unconventional Natural Gas Development and Nasal and Sinus, Migraine Headache, and Fatigue Symptoms in Pennsylvania	Environmental Health Perspectives	Pennsylvania	Cross-sectional	Self-reported symptoms	Chronic rhinosinusitis (CRS), migraine headache, and fatigue symptoms in combination (self-reported): CRS and fatigue, migraine headache and fatigue, and all three symptoms together	NA	Low (5)
**Whitworth [39]**	2017	Maternal Residential Proximity to Unconventional Gas Development and Perinatal Outcomes among a Diverse Urban Population in Texas	PLOS ONE	Texas	Retrospective cohort	Birth outcomes	Preterm birth and fetal death	Small for gestational age and term birth weight	Low (7)
**Whitworth [40]**	2018	Drilling and Production Activity Related to Unconventional Gas Development and Severity of Preterm Birth	Environmental Health Perspectives	Texas	Nested case-control	Birth outcomes	Preterm birth	NA	Low (9)

NA = Not applicable (no result). ^+^ Denotes evidence of a significant negative relationship (i.e., with increasing exposure, poor health outcomes improved). ^a^ High risk pregnancy was an a priori conclusion and is not a direct effect and therefore was not included in a weight of evidence determination. ^b^ Elliot et al. defined the neurologic category to include symptoms of frequent headaches or migraines, dizziness or balance problems, feeling down, difficulties with concentration or memory, difficulty sleeping or insomnia, feeling anxious or nervous, and seizures. Some of these symptoms are traditionally categorized as psychological.

**Table 4:** Summary of the overall weight-of-evidence determinations for each health outcome.

Health Outcome Categories	Total Number of Studies	Health Outcomes	Reference	Number of Studies Per Certainty Rating						Weight of Evidence
				Positive Association			Null Association			
				High	Moderate	Low	Low	Moderate	High	
Birth defects	2	Congenital heart defects	McKenzie [32]			1				Insufficient
		Oral clefts	McKenzie [32]				1			Insufficient
		Neural tube defects	McKenzie [32]			1				Insufficient
		Birth defects prevalence	Ma [33]				1			Insufficient
Birth outcomes	8	Decreased term birth weight or low birth weight	Casey [35]; Currie [36]; Hill [37]; McKenzie [32]; Stacy [38]; Whitworth [39]		2	1	2	1		Mixed
		Early infant mortality	Busby [34]			1				Insufficient
		Fetal death	Whitworth [39]				1			Insufficient
		Gestation period	Hill [37]					1		Insufficient
		Low infant health index	Currie [36]			1				Insufficient
		Low APGAR score ^a^	Casey [35]; Hill [37]		1			1		Mixed
		Preterm/premature birth	Casey [35]; Hill [37]; McKenzie [32]; Stacy [38]; Whitworth [39,40]		1	3	1	1		Mixed
		Small for gestational age	Casey [35]; Hill [37]; Stacy [38]; Whitworth [39]		2		1	1		Mixed
Cancer	3	Cancer incidence (childhood)	Fryzek [47]				1			Insufficient
		Leukemia (childhood non-specific and acute lymphocytic leukemia)	Fryzek [47]; McKenzie [48]			1	1			Mixed
		Non-Hodgkin’s lymphoma (childhood)	McKenzie [48]				1			Insufficient
		CNS tumors^b^(child)	Fryzek [47]			1				Insufficient
		Urinary bladder	Finkel [46]			1				Insufficient
		Thyroid	Finkel [46]				1			Insufficient
		Leukemia	Finkel [46]				1			Insufficient
Cardiovascular	3	Hospitalizations	Jemielita [53]; Peng [54]			1	1			Mixed
		Self-reported symptoms	Rabinowitz [55]				1			Insufficient
Dermal	2	Self-reported symptoms	Elliott [52]; Rabinowitz [55]			1	1			Mixed
Gastrointestinal	2	Self-reported symptoms	Elliott [52]; Rabinowitz [55]				2			Limited- failing to show an association
Neurological	4	Hospitalizations	Jemielita [53]			1				Insufficient
		Self-reported symptoms	Elliott [52]; Rabinowitz [55]; Tustin [57]				3			Limited- failing to show an association
Psychological	2	Self-reported symptoms	Casey [36]; Tustin [57]			1	1			Mixed
		Diagnosed sleep disturbances	Casey [36]			1				Insufficient
Respiratory	6	Self-reported symptoms	Elliott [52]; Rabinowitz [55]; Tustin [57]			1	2			Mixed
		Hospitalizations	Jemielita [53]; Peng [54]			1	1			Mixed
		Asthma exacerbation	Rasmussen [56]		1					Limited
Other	2	Self-reported symptoms (multiple)	Elliott [52]; Tustin [57]			2				Limited
		Hospitalizations (all)	Jemielita [53]				1			Insufficient

^a^ APGAR score: Appearance, Pulse, Grimace, Activity and Respiration score. ^b^ CNS: Central Nervous System.

## Supplementary Materials

The following materials are available online at https://www.mdpi.com/1660-4601/16/12/2123/s1, Tables S1–S20: Study evaluation individual assessments, Table S21: Full summary details of epidemiologic studies included in systematic review, Table S22: Summary of answers to study evaluation questions.
